# Analysis of thromboembolic events in patients with non-small cell lung cancer who received adjuvant chemotherapy: single-center real-world data

**DOI:** 10.1038/s41598-022-11631-9

**Published:** 2022-05-10

**Authors:** Tae-Hwan Kim, Yong Won Choi, Hyun Woo Lee, Seok Yun Kang, Heejun Son, Jin-Hyuk Choi, Mi Sun Ahn, Seung-Soo Sheen

**Affiliations:** 1 Department of Hematology-Oncology, Ajou University School of Medicine, 164, World cup-ro, Yeongtong-gu, Suwon, 16499 Gyeonggi-do Korea; 2 Department of Pulmonology and Critical Care Medicine, Ajou University School of Medicine, Suwon, Korea

**Keywords:** Cancer, Oncology

## Abstract

Thromboembolic events (TEEs) are common in cancer patients, with increased risk of TEE by chemotherapy in patients with lung cancer. However, TEEs in patients with non-small cell lung cancer (NSCLC) who received adjuvant chemotherapy have rarely been reported. This study retrospectively analyzed real-world data of 275 patients with NSCLC treated with adjuvant chemotherapy after surgery from October, 2005 to June, 2020, in a single institution. The incidence of TEEs during or within one year of completion of adjuvant chemotherapy was investigated, and factors related to TEEs were analyzed. TEEs were confirmed in nine patients (3.3%), without fatal event related to TEEs. None of the factors, including Khorana score, was significantly associated with the occurrence of TEEs. All patients with TEEs had pathologic stage IIB or higher and a history of smoking, except for one patient. In conclusion, TEEs occurred in a smaller proportion of patients with NSCLC treated with adjuvant chemotherapy in the real world compared with those treated with palliative chemotherapy in previous reports. Furthermore, prophylactic anticoagulation in patients with NSCLC receiving adjuvant chemotherapy may not be needed except for high-risk patients, although those patients should be informed about the possible risk of TEEs.

## Introduction

Lung cancer is the second most frequent newly diagnosed malignancy with the highest mortality rate worldwide^1^. In non-small cell lung cancer (NSCLC), which comprises approximately 87% of newly diagnosed cases, complete surgical resection is still the mainstay of treatment for operable disease^2–6^. Moreover, platinum-based adjuvant chemotherapy has been established as the standard management for patients with postoperative stage II/III or high-risk IB NSCLC^3–6^.

A thromboembolic event (TEE) is a relatively common problem in cancer patients with variable causes^7,8^. In addition, the risk of TEE can increase with the administration of chemotherapy^9–11^. TEEs have been reported in patients with NSCLC receiving palliative chemotherapy or those with small cell lung cancer (SCLC) in several studies^9,12–20^. However, there were very few reports about TEEs in NSCLC patients who underwent adjuvant chemotherapy. Therefore, we aimed to retrospectively analyze real-world data about TEEs in patients with NSCLC who underwent adjuvant chemotherapy after surgery.

## Methods

### Patients

All patients with histologically confirmed postoperative pathologic stage IB to III NSCLC who initiated adjuvant chemotherapy following surgical resection between October 2005 and June 2020 at Ajou University Hospital, Suwon, Korea, were retrospectively identified. Patients with gross residual lesions after surgery, patients who had undergone preoperative chemotherapy or radiation therapy, and patients who had a history of TEEs within three months before the start of adjuvant chemotherapy were excluded. Patients with a history of chemotherapy for lung or other types of cancers were included, except those who had previously received platinum agents (cisplatin, carboplatin, oxaliplatin).

All procedures in the study involving human participants were carried out in accordance with the ethical standards of the institutional and/or national ethical committee and with the 1964 Helsinki Declaration and its later amendments or comparable ethical standards. This research protocol was approved by the institutional review board (IRB) of Ajou University Hospital (IRB approval no. AJIRB-MED-MDB-21-315). The institutional review board of Ajou University Hospital waived the need of informed consent because this study was conducted using the medical records of anonymized patients.

### Clinical review

We retrospectively reviewed the medical records of eligible patients and collected their data, such as age, gender, body mass index, Eastern Cooperative Oncology Group (ECOG) performance status, smoking status, adjuvant chemotherapeutic regimen, status of adjuvant radiotherapy, histologic type, pathologic stage according to American Joint Committee on Cancer 8th edition^21^, presence of diabetes mellitus, presence of hypertension, history of vascular disease, history of other malignancies, Khorana score, presence of TEE within one year after the completion date of adjuvant chemotherapy, and types of TEE. A TEE was defined as any type of thrombosis, including venous thrombosis such as deep vein thrombosis and pulmonary embolism, and arterial thrombosis, such as myocardial infarction and cerebral infarction. TEE was confirmed by radiological imaging tests, such as computed tomography (CT), magnetic resonance imaging, Doppler ultrasonography, and coronary angiography.

In the current study cohort, chest CT was not routinely performed before the initiation of adjuvant chemotherapy except planning CT for patients who underwent radiotherapy prior to chemotherapy. Patients were usually followed with chest CT every 3 months for at least one year after completion of adjuvant chemotherapy. Other imaging tests except plain chest X-ray were performed only when clinically indicated.

Khorana score was calculated on the basis of primary tumor site, pre-chemotherapy white blood cell and platelet count, hemoglobin level, and body mass index^22^.

### Statistical analysis

Fisher’s exact test was used to determine the factors influencing the occurrence of TEEs with p-values of < 0.05. Statistical analyses were two-sided and performed using IBM SPSS Statistics software version 25.

## Results

### Patient characteristics

A total of 279 patients with NSCLC who underwent adjuvant chemotherapy after surgery were identified within the defined period. Among these patients, three who had a history of TEE within three months before the start of adjuvant chemotherapy and one who had previously undergone cisplatin-containing chemotherapy for extranodal NK-/T-cell lymphoma were excluded, leaving 275 patients for analysis.

The patient characteristics are summarized in Table 1. A total of 231 patients (84.2%) were under 70 years of age, and male patients predominated (194 patients, 70.5%). Among the patients, 197 patients (71.6%) had a body mass index of less than 25 kg/m^2^, and most had an ECOG performance status of 0 (*n* = 190, 69.1%) or 1 (*n* = 83, 30.2%). Furthermore, 195 patients (70.9%) had a history of smoking, and chemotherapeutic regimens were vinorelbine/cisplatin (*n* = 148, 53.8%), paclitaxel/carboplatin (*n* = 119, 43.3%), and other regimens (*n* = 8, 2.9%; etoposide/cisplatin: 5, vinorelbine/carboplatin: 2, gemcitabine/carboplatin: 1). The median time interval between surgery and the start of adjuvant chemotherapy was 45 days (11–175 days). Adjuvant radiotherapy was performed in 117 patients (42.5%, before chemotherapy: 82 patients, after chemotherapy: 35 patients). Adenocarcinoma was the most common histologic type (*n* = 188, 68.4%), and pathologic stages were identified as IB (*n* = 39, 14.2%), IIA (*n* = 12, 4.4%), IIB (*n* = 89, 32.4%), IIIA (*n* = 103, 37.5%), and IIIB (*n* = 32, 11.5%).

**Table 1:** Patient characteristics.

Clinical characteristics	Total (*n* = 275)	Non-TEE (*n* = 266)	TEE (*n* = 9)	p-value*
**Age, years**				
< 70	231 (84.0%)	224 (84.2%)	7 (77.8%)	0.640
≥ 70	44 (16.0%)	42 (15.8%)	2 (22.2%)	
**Gender**				
Male	194 (70.5%)	187 (70.3%)	7 (77.8%)	1.000
Female	81 (29.5%)	79 (29.7%)	2 (22.2%)	
**BMI**				
< 25	197 (71.6%)	190 (71.4%)	7 (77.8%)	1.000
≥ 25	78 (28.4%)	76 (28.6%)	2 (22.2%)	
**ECOG PS**				
0	190 (69.1%)	184 (69.2%)	6 (66.7%)	1.000
1	83 (30.2%)	80 (30.0%)	3 (33.3%)	
2	2 (0.7%)	2 (0.8%)	0 (0.0%)	
**Smoking**				
Never	80 (29.1%)	79 (29.7%)	1 (11.1%)	0.455
Current or former	195 (70.9%)	187 (70.3%)	8 (88.9%)	
**Chemotherapeutic regimen**				
VP	148 (53.8%)	143 (53.8%)	5 (55.6%)	1.000
TC	119 (43.3%)	115 (43.2%)	4 (44.4%)	
Others	8 (2.9%)	8 (3.0%)	0 (0.0%)	
**Adjuvant radiotherapy**				
No	158 (57.5%)	154 (57.9%)	4 (44.4%)	0.502
Yes	117 (42.5%)	112 (42.1%)	5 (55.6%)	
**Histology**				
Adenocarcinoma	188 (68.4%)	183 (68.8%)	5 (55.6%)	0.146
Squamous cell carcinoma	70 (25.5%)	68 (25.6%)	2 (22.2%)	
Others	17 (6.2%)	15 (5.6%)	2 (22.2%)	
**Stage**				
IB	39 (14.2%)	39 (14.7%)	0 (0.0%)	
IIA	12 (4.4%)	12 (4.5%)	0 (0.0%)	0.630
IIB	89 (32.4%)	84 (31.6%)	5 (55.6%)	
IIIA	103 (37.5%)	100 ( 37.6%)	3 (33.3%)	
IIIB	32 (11.5%)	31 (11.6%)	1 (11.1%)	
**Diabetes mellitus**				
No	220 (80.0%)	212 (79.7%)	8 (88.9%)	0.693
Yes	55 (20.0%)	54 (20.3%)	1 (11.1%)	
**Hypertension**				
No	195 (70.9%)	188 (70.7%)	7 (77.8%)	1.000
Yes	80 (29.1%)	78 (29.3%)	2 (22.2%)	
**History of vascular disease**^**†**^				
No	257 (93.5%)	248 (93.2%)	9 (100.0%)	1.000
Yes	18 (6.5%)	18 (6.8%)	0 (0.0%)	
**Khorana score**				
1	222 (80.7%)	214 (80.5%)	8 (88.9%)	
2	50 (18.2%)	49 (18.3%)	1 (11.1%)	1.000
3	2 (0.7%)	2 (0.8%)	0 (0.0%)	
4	1 (0.4%)	1 (0.4%)	0 (0.0%)	
**Time interval between surgery and the start of adjuvant chemotherapy**				
< 45 days^‡^	137 (49.8%)	135 (50.8%)	2 (22.2%)	0.172
≥ 45 days	138 (50.2%)	131 (49.2%)	7 (77.8%)	

*TEE* thromboembolic event, *BMI* body mass index, *ECOG* Eastern Cooperative Oncology Group, *PS* performance status, *VP* vinorelbine/cisplatin, *TC* paclitaxel/carboplatin.

*Fisher’s exact test.

^†^Types of vascular disease in the patients were identified as either coronary arterial occlusive disease or cerebrovascular disease.

^‡^Median.

A total of 55 (20.0%), 80 (29.1%), and 18 (6.5%) patients had history of diabetes mellitus, hypertension, and vascular disease, respectively, and the patients with Khorana score of 1 predominated (*n* = 222, 80.7%). Sixteen patients (5.8%) had previous history of other types of malignancies (gastric cancer: 7, others: 9 [one type each]), without prior diagnosis of lung cancer. Among them, 6 patients had history of chemotherapy (adjuvant chemotherapy for gastric cancer [duodenal cancer]: 4 [1], chemotherapy for lymphoma with complete remission status: 1), while one patient had been treated with androgen deprivation therapy for prostate cancer with complete remission status. In addition, one patient had previously received intravesical BCG for bladder cancer.

### Analysis of the patients with TEE

TEEs were confirmed in 9 patients (3.3%), with one patient identified with TEE after recurrence. The median time interval of TEE occurrence from surgery and that from the initiation of chemotherapy were 205 days (116–428 days) and 119 days (47–406 days), respectively. All analyzed clinical factors were not significantly associated with the occurrence of TEE (Table 1), although eight out of nine patients with TEE (89%) had a smoking history (current smoker: 5, former smoker: 3). TEE was not identified in patients with stage IB or IIA NSCLC, and most patients had Khorana score of 1 (*n* = 8, 89%). There was no episode of TEEs in 16 patients with previous history of chemotherapy.

In terms of the types of TEEs, 7 patients (78%) had pulmonary thromboembolism, one (11%) had pulmonary venous thrombosis, and one (11%) had internal jugular venous thrombosis (Table 2). All TEEs were diagnosed after the completion of chemotherapy, although no fatal event related to TEE was found. Among 8 patients without recurrence at the time of TEE, 6 patients were identified with TEE within three months after completion of adjuvant chemotherapy. Twenty-three patients (8.4%) received perioperative thromboprophylaxis for less than one week with low molecular weight heparin (dalteparin: 21, enoxaparin: 2) without episode of TEE after chemotherapy. In addition, no patient underwent anticoagulant therapy at the time of initiation of adjuvant chemotherapy.

**Table 2:** Types of thromboembolic events in patients with non-small cell lung cancer who received adjuvant chemotherapy.

Type of thromboembolic events	Total (n = 9)
Pulmonary thromboembolism	7 (78%)
Pulmonary venous thrombosis	1 (11%)
Internal jugular venous thrombosis	1 (11%)

## Discussion

To the best of our knowledge, no real-world study has been reported about TEEs in patients with NSCLC receiving adjuvant chemotherapy. The incidence of TEE was identified as 3.3% in this study without any significant clinical factor associated with the risk of TEE.

Multiple causes, such as an overexpression of tissue factor, thrombocytosis, leukocytosis, anemia, infection, old age, circulatory stasis, and immobilization, may contribute to the occurrence of TEE in patients with cancer^10,23–26^. Furthermore, the administration of anti-cancer drugs, especially platinum compounds, the essential agents for adjuvant chemotherapy for NSCLC, is associated with the risk of TEE^9–12,18,25,26^. Although the mechanisms of platinum-induced hypercoagulability are not entirely clear, several reports have suggested possible pathophysiologic processes such as vascular endothelial cell damage and necrosis, platelet activation and aggregation, and increased procoagulant activity^11,27,28^.

In a defined urban population, the incidence of venous thromboembolism has been reported to be 1.6 per 1000 patients (0.16%) in a year^29^. In previous studies, the incidence of TEEs varies from 0% in patients with stage IB and II NSCLC without adjuvant chemotherapy after surgery to 13.6% in patients with NSCLC including all stages^3,9,30–32^. The incidence of TEEs was reported to be 7.9–17.6% in patients with NSCLC treated with platinum-based chemotherapy, mostly those with stage III or IV^9,14,17,18^. Furthermore, TEEs were confirmed in 10.2% of patients with SCLC treated with first-line platinum-based doublet chemotherapy^20^. Taken together, the incidence of TEEs seems to be lower in patients with NSCLC treated with adjuvant chemotherapy than those with advanced NSCLC or those with SCLC receiving chemotherapy. The absence of gross tumor burden may be attributed to the relatively lower risk of TEE in patients with NSCLC undergoing adjuvant chemotherapy^26^.

No significant factor related to the occurrence of TEEs was found in this study. Statistically significant findings might not have been observed due to the low incidence (3.3%) in a total of 275 patients, although a study with larger sample size may reveal some factors associated with TEE. Khorana score^22^, previously known to be related to the occurrence of TEEs, was not a risk factor for TEE, mainly because eight (89%) of the patients with TEE had Khorana score of 1. In addition, diabetes mellitus and hypertension did not correlate with the incidence of TEE as previously reported, whereas radiotherapy and history of vascular disease, which are known to be associated with the occurrence of TEEs, were not found to be significant factors^12,14,19,31,32^. Although all TEEs occurred in patients with NSCLC of stage IIB or higher, whether a higher stage is a risk factor for the occurrence of TEEs was difficult to determine, considering the absence of statistical significance. In addition, no significant difference was found in the incidence of TEEs between vinorelbine/cisplatin and paclitaxel/carboplatin, which are standard regimens in adjuvant chemotherapy for NSCLC^33^. Nonetheless, the result that all patients except one with TEEs had a smoking history seems to be clinically meaningful, although statistical significance was not observed. This is consistent with previous reports demonstrating a relationship between smoking history and the occurrence of TEEs in patients with stage IV NSCLC or SCLC receiving chemotherapy^20,32^. Therefore, a more detailed explanation about TEE risk seems to be needed for patients with a smoking history before starting chemotherapy. In addition, treating physicians should be aware of possible TEE occurrence with more careful monitoring for these patients during or for a certain period of time after chemotherapy.

Two phase III trials (the AVERT and CASSINI) investigated the role of thromboprophylaxis with direct oral anticoagulants in patients with various types of cancer receiving chemotherapy with conflicting results^34,35^. Unlike the current study including majority of patients with Khorana score 1 (about 80%), almost all patients enrolled in both trials had Khorana score 2 or higher. In addition, although no patient received prophylactic anticoagulation at the start of chemotherapy in the present study, the incidence of TEE was even lower than that of patients treated with direct oral anticoagulants in the two trials (4.2% and 6.0%, respectively). Therefore, prophylactic anticoagulation in NSCLC patients receiving adjuvant chemotherapy may not be recommended except for high-risk patients.

This study has some limitations. First, given that it was a retrospective analysis with possible selection bias, TEE incidence might have been underestimated. For example, TEE diagnosed at other hospitals might not be identified. Second, it was difficult to determine the factors related to TEE with statistical significance because the absolute number of patients with TEE was small. Third, asymptomatic patients with TEE might not be identified because imaging tests were performed only when clinically indicated, although chest CT was usually followed every three months. Finally, the current study cohort comprised patients from a single institution over a fairly long period. Nevertheless, to our knowledge, the only report about the incidence of TEEs in adjuvant chemotherapy for NSCLC is the JBR.10 phase III trial in patients with stage IB or II NSCLC^3,9^. In the trial, the incidence of TEE was 2.9% in patients treated with vinorelbine/cisplatin chemotherapy, which is almost comparable to the result of current study^3,9^. Therefore, the present study seems to be clinically relevant, given that it investigated all NSCLC patients treated with adjuvant chemotherapy within the defined period in a real-world setting, demonstrating a relatively lower incidence of TEEs compared with that in patients with NSCLC receiving platinum-based palliative chemotherapy.

In conclusion, TEEs occurred in a smaller proportion of patients with NSCLC treated with adjuvant chemotherapy in the real world compared with those treated with palliative chemotherapy in previous reports. Furthermore, prophylactic anticoagulation in patients with NSCLC receiving adjuvant chemotherapy may not be needed except for high-risk patients, although those patients should be informed about the possible risk of TEEs.

## Data Availability

The datasets generated and/or analyzed during the current study are not publicly available due to the confidentiality of the data of patient but are available from the corresponding author on reasonable request.
